# Risk factors of 90-day rehospitalization following discharge of pediatric patients hospitalized with mycoplasma Pneumoniae pneumonia

**DOI:** 10.1186/s12879-019-4616-9

**Published:** 2019-11-12

**Authors:** Le Wang, Zhishan Feng, Jinfeng Shuai, Jianhua Liu, Guixia Li

**Affiliations:** 1 Institute of Pediatric Research, Children’s Hospital of Hebei Province, 133 Jianhua South Street, Shijiazhuang, 050031 Hebei Province China; 2 Hebei General Hospital, Shijiazhuang, 050000 China; 3 No.2 Department of Respiratory, Children’s Hospital of Hebei Province, 133 Jianhua South Street, Shijiazhuang, 050031 Hebei Province China

**Keywords:** MPP, Rehospitalization, Children

## Abstract

**Background:**

Among pediatric patients hospitalized for Mycoplasma pneumoniae pneumonia (MPP), the risk factors for 90-day readmission after discharge is undefined.

**Methods:**

We conducted a retrospective observational study of patients <14 years of age who were discharged with a diagnosis of MPP between January 2016 and February 2017. We collected clinical, laboratory and radiographic variables at the time of initial admission. We assessed pneumonia-related readmission within 90-day after discharge. Risk factors independently associated with rehospitalization were identified using multiple logistic regression models.

**Results:**

Of the 424 MPP hospitalizations, 48 (11.3%) were readmitted within 90 days and were mainly diagnosed with pneumonia. Patients with younger age or coinfection with influenza A were more likely to be readmitted. In addition, compared with children without readmission, the readmission ones showed different clinical and laboratory characteristics at the index hospital admission. Multiple logistic regression analysis identified age (OR 0.815, 95%CI 0.706–0.940) and body temperature (OR 0.659, 95%CI 0.518–0.839) were significantly associated with lower risk of 90-day readmission. Coinfection with influenza was independently associated with a greater likelihood of 90-day readmission (OR 4.746, 95%CI 1.191–18.913).

**Conclusions:**

Readmission after MPP are common and is related to patients’ age, body temperature and influenza A coinfection during initial hospital stay, indicating potential targets could be noticed to reduce the rehospitalization after pediatric MPP.

## Introduction

Readmission of patients initially hospitalized for community acquired pneumonia (CAP) is relatively common [1–3]. Both of preventable and non-preventable risk factors have been explored, but the main participants in these studies were the elderly and patients with multiple comorbidities, not children [4–7].

The previous three studies described 14 days [8], 30 days [9] readmission rates (range of 1.5–8%) for children with pneumonia or lower respiratory infections (LRIs) [10]. In these studies, one of the consistent identified risk factors was chronic medical conditions. But in fact, a large number of children who are rehospitalized are caused by acute diseases [11]. If patients with chronic conditions are excluded, the difficulty is to detect readmission risk factors associated with the current acute infection. In addition, children with LRIs, a relatively broad diagnosis, or pneumonia with an underlying chronic illness may bring compounding factors conferring susceptibility for readmission. A promising approach to resolve this problem is to narrow down study sample according to the pathogenic or clinical features, such as Mycoplasma pneumoniae pneumonia (MPP), which accounts for up to 40% pediatric CAP [12], and its diagnosis is based on etiology and clinical evidence, thereby elevating the power to detect readmission risk factors associated with the current acute infections. We hypothesized that MPP children may have different characteristic during the first hospital stay between patients with and without readmission. This study was therefore conducted to enroll pediatric MPP patients without other underlying chronic diseases.

Our aims were to (1) describe the incidence and type of readmission after MPP discharge, (2) investigate the differences between patients with and without readmission at the initial hospital stay, (3) examine the risk factors for 90-day pneumonia-related rehospitalization.

## Material and methods

### Study design

This retrospective, observational study was conducted at Children’s Hospital of Hebei Province, a 1200-bed teaching hospital in Hebei Province (northern China) that serves a population of 70,000,000 inhabitants, including 18.5% children. Patients with a discharge diagnosis of MPP were evaluated. The project was approved by the ethics review board of the hospital. Because data in this report were collected from inpatient electronic medical records, there was no need to collect new specimens or the corresponding written informed consent.

### Study sample

Children ≤14 years of age who were admitted to Children’s Hospital of Hebei Province with a diagnosis of MPP from January 2016, to February 2017, were consecutively enrolled into the study. The diagnosis of MPP needs to meet the following first 2 points plus either third or fourth point [13]: 1) a new infiltrate on a chest radiograph; 2) fever, cough and abnormal lung auscultation; 3) positive serology laboratory results specific MP antibody titer≥1:160 detected by a micro-particle agglutination test [14]; 4) positive PCR laboratory results, MP-DNA positive detected in sputum, nasopharyngeal aspirate or bronchoalveolar lavage fluid (BALF) by PCR [15]. Patients were excluded from the study if they were known to be chronically immunosuppressed, or with chronic cardiopulmonary conditions or had been hospitalized for the previous 14 days. If a patient had more than one episode of pneumonia during the index hospitalization, only the first one was included in the analysis.

### Data and specimen collection

The following patient characteristics were evaluated: age, sex, signs and symptom before admission (wheezing, cough and diarrhea). The laboratory data and radiological findings were also measured and retrospectively investigated from inpatient electronic medical records system. Clinical symptoms included wheezing, cough and diarrhea. Ill day and febrile day before admission, hospital days, febrile day and readmission rate were also recorded. The time frame of readmission was set as 90-day from original discharge. Body temperature was examined at the beginning of admission and every 8 h thereafter. A febrile day was defined as the body temperature exceeded 38.0 °C at least once [16].

Patients were asked to cough, and the expectorated sputum was collected. If the child is too young to cough, a sterile negative pressure suction catheter is applied to obtain the oropharyngeal suction (OPS). The storage, transportation and nucleic acid extraction procedure were reported elsewhere [17]. The paired serum samples were taken at the presentation of pneumonia and at least 7 days after the first collection of serum. The serum was obtained from 2 mL whole blood by the separation gel tube.

### Detection of MP-DNA and MP-antibody

The GeXP assay (GenomeLab GeXP Genetic Analysis System) was performed on all specimens for the 13 type/subtypes of common respiratory pathogens including *M. pneumoniae*. The multiplex-PCR was performed as previously described elsewhere [18]. The bacteria infection was examined by standard culture methods from sputum specimens [19]. The determination of MP-specific antibody was performed using a commercially available micro-particle agglutination test Serodia-MycoII kit (Fujirebio, Tokyo, Japan) [14]. Diagnosis criteria were defined as ≥4-found rising for paired sera or single serum of titer ≥1:160 [15].

### Statistical analysis

The chi-squared test was used to compare categorical variable in subgroups. And for those continuous variables with normal and non-normal distributions, mean or median values were compared using the t test or Mann-Whitney U test. SPSS 19.0 statistics package (SPSS Inc., Chicago, USA) software was used for all statistical analysis. *p* < 0.05 was considered statistically significant.

## Results

### Patients enrollment and readmission diagnoses

**Fig. 1:**
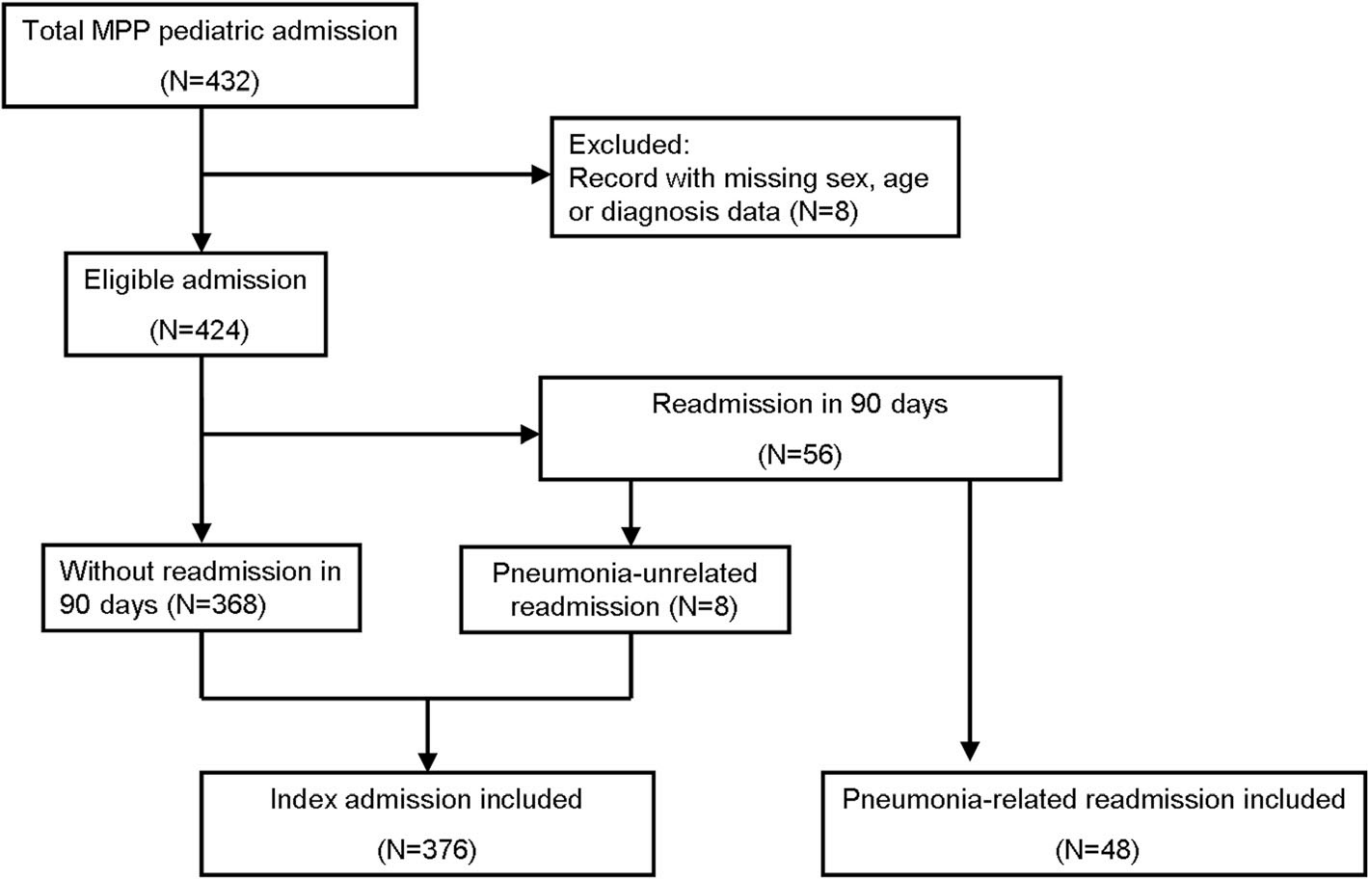
Patient flow chart, the index admission and 90-days readmission. The diagnosis of pneumonia-unrelated readmission included: 3 epilepsy, 2 encephalitis, 2 carditis and 1 arthrosynovitis cases

During the study period, 432 inpatients met our study eligibility criteria for MPP: 8 patients were excluded due to the missing sex, age or diagnostic data, and 368 patients were not readmitted to hospital within 90 days (Fig. 1). Of the 56 patients who were readmitted in 90 days, 8 were diagnosed with pneumonia-unrelated diseases including epilepsy, encephalitis, carditis and arthritis. Therefore, 48 (11.3%) children were readmitted due to pneumonia-related diseases in 90 days of initial MPP discharge. Of the 48 re-admitted cases, 17 (35.4%) were reinfected with MP, 23 (47.9%) were negative for MP, and 8 (16.7%) did not receive pathogen detection test. The most common cause of readmission was pneumonia (47.9%), followed by bronchial pneumonia (45.8%) bronchitis (4.2%) and one (2.1%) refractory mycoplasma pneumoniae pneumonia (RMPP) (Table 1). Among the enrolled 424 patients, there were 23 cases were detected to be positive by PCR alone, 129 were serology alone (with 6 cases as seroconversion and 123 cases as single high titer) and 272 were positive by both PCR and serology assays.

**Table 1:** The diagnosis of 90-days pneumonia-related readmission

Readmission diagnosis	No.	%
Pneumonia	23	47.9
Bronchopneumonia	22	45.8
Bronchitis	2	4.2
Refractory Mycoplasma pneumoniae pneumonia	1	2.1

### Characteristics of children

Demographic characteristics included age (median age 4.5, range in 0.1–13) and gender (238 boys and 186 girls). As shown in Table 2, patients who were readmitted in 90 days were significantly younger than those without readmission (median age: 2 v.s. 4.7 years, *p* < 0.001) and were more likely to show wheezing symptoms (*p* < 0.001), as well as a lower body temperature on admission (*p* < 0.001), shorter febrile days during hospital stay (*p* < 0.001). Similarly, more patients with readmission showed normal (*p* = 0.009) or light diffuse shadowing (*p* = 0.030) radiological findings. CRP, LDH, HBDH and neutrophil percentage levels were lower, but the percentage of lymphocyte was significantly greater (all *p* < 0.05) (Table 3).

**Table 2:** Patient characteristic on index visit are different between children with and without readmission

		With readmission	Without readmission	*P*
		*n* = 48	*n* = 376	
Sociodemographic	Sex (Male)	31 (64.6%)	211 (56.1%)	0.268
	Age (Year)	2 (1–4.6)	4.7 (2.5–7)	<0.001
Before and after admission	Ill day before admission	10 (5.5–15)	8 (6–14)	0.449
	Febrile day before admission	3 (0–8.5)	6 (3–10)	0.002
	Body Temperature on admission	38.5 (36–39.4)	39.3 (38.6–39.8)	<0.001
	Hospital day	9 (7–15)	10 (7–14)	0.819
	Febrile day after admission	1 (0–3)	2 (0–4)	<0.001
Clinical manifestation at presentation	Wheezing day	5 (3–8)	3 (2–10)	<0.001
	Cough day	9 (4.5–15)	7 (5–12)	0.532
	Diarrhea day	6 (5–7)	2.5 (2–3)	0.397
Radiographic finding	Normal	11 (22.9%)	38 (10.1%)	0.001^a^
	Light diffuse shadowing	15 (31.2%)	68 (18.1%)	
	Consolidation	16 (33.3%)	169 (44.9%)	
	Pleural effusion	5 (10.4%)	75 (19.9%)	
Severity on admission	ICU admission	7 (14.6)	26 (6.9%)	0.114
	Mechanical ventilation	3 (6.3%)	13 (3.5%)	0.580

Data were shown in median (IQR) or N (%). Comparative analysis was performed using the Chi-Square or Mann-Whitney U test, as appropriate

^a^Separately, *p* values are 0.009 for Normal group, 0.030 for Light diffuse shadowing group, 1.127 for Consolidation group, 0.112 for Pleural effusion group

**Table 3:** Different laboratory data on index hospitalization between patients with and without readmission

Laboratory data	With readmission	Without readmission	*P*
	*n* = 48	*n* = 376	
WBC (10^9/L)	8.6 (7.3,11.3)	9.9 (7.6, 13.2)	0.192
Neutrophil (%)	49.2 ± 17.5	55.6 ± 16.3	0.024
Lymphocyte (%)	40.4 ± 16.0	31.9 (21.3, 43.6)	0.008
Monocyte (%)	7.1 ± 2.6	6.6 (5.4–8.2)	0.637
CRP (mg/L)	4.2 (1.0, 11.4)	10.4 (1.4–30.0)	0.025
LDH (IU/L)	261.5 (228.7, 299.2)	290.0 (244.7–373.0)	0.009
HBDH (IU/L)	211.5 (189.0, 236.2)	237.5 (201.7–296.0)	0.005

*WBC* White blood cell, *LDH* Lactatedehydrogenase, *HBDH* Hydroxybutyrate dehydrogenase, *CRP* C-reactive protein

Data were shown in mean ± SD or median (IQR)

Comparative analysis was performed using Chi-Square or Mann-Whitney U test

### Coinfection pathogens

Coinfection was observed in 189 (44.6%) cases, and children infected with influenza A were more likely to be admitted again (*p* < 0.001, Table 4). Coinfection rates of other pathogens (rhinovirus, parainfluenza, influenza B, respiratory syncytial virus, adenovirus, coronavirus, human metapneumovirus, *S. pneumoniae* and human bocavirus) did not vary significantly between patients with and without rehospitalization.

**Table 4:** The coinfection pathogens in patients with 90-days readmission

Pathogen	No.^a^	Readmission cases	*P*
Rhinovirus	80	12 (15.0%)	0.249
Parainfluenza	33	5 (15.2%)	0.470
Influenza B	29	5 (17.2%)	0.297
Respiratory syncytial virus	17	1 (5.9%)	0.740
Adenovirus	15	2 (13.3%)	1.000
Coronavirus	13	1 (7.7%)	0.675
Human metapneumovirus	13	1 (7.7%)	1.000
Influenza A	11	6 (54.5%)	<0.001
*S. pneumoniae*	10	1 (10.0%)	0.894
Human Bocavirus	6	0 (0.0%)	0.816

^a^There are mixed infections with more than one pathogen, the total cases is not equal to 189. There are 155 cases of single pathogen coinfection, 30 cases of two pathogens, 4 cases of 3 pathogens

### Multiple logistic regression analysis

Multiple logistic regression analysis identified age (OR 0.815, 95%CI 0.706–0.940) and body temperature (OR 0.659, 95%CI 0.518–0.839) during initial hospital stay were significantly associated with lower risk of 90-day readmission. Coinfection with influenza A at index admission was independently associated with a greater likelihood of 90-day readmission (OR 4.746, 95%CI 1.191–18.913) (Table 5).

**Table 5:** Stepwise logistic regression for the related factors associated with readmission

Positive Variables	*P*-value	OR	95% CI	
			Lower	Upper
Age	0.005	0.815	0.706	0.940
Influenza A coinfection	0.027	4.746	1.191	18.913
Body Temperature on admission	0.001	0.659	0.518	0.839

## Discussion

Generally, readmissions after pneumonia are common. In terms of safety and cost, it is important to assess the relationship between initial hospital stay and readmission outcomes [9, 20]. Although the investigation of risk factors is challenging, significant progress has been made on the elderly, that the readmission was found to be largely depends on the comorbidities and factors external to the patient [1, 2, 4, 21]. This has also been observed in children, and one of the identified risk factors is chronic medical conditions such as underlying pulmonary or cardiovascular disease [9–11]. To date, research on potential risk factors for readmission has hardly focused on current acute infections or specific pathogens. Because pneumonia is a complex heterogenous disease that can be caused by a variety of pathogens, 1) studying pneumonia patients with the same infectious pathogen can reduce heterogeneity, 2) exclusion of patients with underlying diseases can improve the ability to detect readmission risk factors associated with the current acute infections. Therefore, we investigated the rate of 90-day pneumonia-related readmission in hospitalized children with MPP who had no basal or chronic disease. After comparing the clinical information of the first hospitalization between patients who were readmitted and not readmitted to the hospital, we obtained the following findings: 1) 48 (11.3%) children were readmitted within 90 days of the first MPP discharge; 2) at the index hospital stay, readmission patients manifested different characteristics; 3) co-infection with influenza A increased the risk of 90-day readmission.

In this study, the readmission rate after MPP discharge was 11.6%. Pneumonia and bronchial pneumonia were the majority diagnoses on readmission. Nakamura et al. identified that 5.5% cases readmitted after LRI hospitalization, and the most common readmission diagnose was LRI (48.2%) [10]. Similar to the pattern observed by Neuman and colleagues, nearly half of the 8% of patients who were discharged from initial pneumonia hospitalization were also associated with pneumonia [9]. Previous work has shown that 30% pediatric readmissions are potentially preventable, especially the index admission and readmission are causally related [22]. This is one of the reasons we focus on pneumonia-related readmission. Meanwhile, we observed a trend in patients with relatively mild radiological symptoms and lower levels of acute inflammatory markers that are more likely to be rehospitalized. The cause of this phenomenon may be related to two points, including different host immunity and treatment strategies. First, clinical presentation depends on the host’s immune response, rather than direct microbial destruction during the progression of *M. pneumoniae* infection [23]. Patient with a reduced immune system, such as younger ones who have had less time to develop immunity, may be characterized by mild clinical symptoms but with a prolonged recovery period. Second, pediatricians will adopt different treatment strategies for patients with severe or mild symptoms. Patients at risk of readmission may receive different medication times due to *M. pneumoniae* virulence or host immune response.

In this present study, we found that influenza coinfection increased the risk of readmission, which is consistent with previous investigations of children with complicated pneumonia. William et al. found that although there was a trend to increase mortality, patients with flu coinfection were less likely to readmitted in 2 weeks readmission, [24]. Brogan et al. observed that children who were infected with influenza during the initial hospital stay had a higher rate of readmission than children who were not infected with influenza [25]. Regarding the elderly, researches show that influenza vaccination is associated with a lower likelihood of readmission [5, 26, 27]. In view of these findings, influenza vaccination should be promoted not only in pediatric hospitals at CAP discharge, but also for all people, particularly in high risk groups including children under 5 years old, and those with asthma. In addition, we observed that younger children are liable to readmit, which is consistent with previous findings, demonstrating a higher rate of readmission for children under 1 year of age [9, 28]. As explained by Gay JC et al., pneumonia in young patients usually has protracted and waning course, leading to structural lung damage or immune paresis and further pneumonia episodes [28]. Second, younger patients may be more prone to new infections due to higher exposure during nursery attendance and the previous lack of immunity to respiratory pathogens, which will be resulting in rehospitalization. Furthermore, Studies of children with asthma have found that the rate of readmission of children under 1 year of age in higher, further highlighting the need to improve inpatient decision-making for young patients.

To our knowledge, this is the first study to explore the factors of readmission for pediatric MPP patients. Further research in larger cohorts is needed to validate the data. Meanwhile, some questions remain to be answered: first, it has been reported that pneumonia attributed to potentially antibiotic-resistant bacteria is associated with an increased risk of readmission [21], we strongly felt that macrolide resistance has a role on the risk of readmission, but what is the role? Second, coinfection with influenza A will increase the risk of readmission, what is the underlying mechanism?

## Limitations

This study has several limitations. First, the sample size may be small because only 48 patients were rehospitalized within 90 days. Second, if the child is readmitted to another institution, we may underestimate the rate of readmission. Third, in the absence of the information after first discharge, the interference factor related to age may be introduced into this research, as the rehospitalization may be caused by a new infection during nursery attendance. Fourth, other clinical information to document severity is not included in the study, such as oxygen requirement, antibiotic or corticoid duration. Fifth, a potential limitation would be that the serological assays have a high false positive detection rate and it is difficult to obtain the second serum. In our report, only 6 children provided paired sera, and the other 123 patients (123/424, 29%) had positive serological results with only a single high titer, but the PCR results were negative. Last, although there were significant differences in CRP, LDH levels or patient characteristics between rehospitalized and non-rehospitalized patients, these factors cannot be controlled and of low value in clinical practice.

## Conclusions

In conclusion, rehospitalization after MPP is relatively common and is related to patients’ age and co-infected pathogens. Careful attention to clinical variables may reduce the frequency of rehospitalization of pediatric patients after discharge on MPP.

## Data Availability

The datasets generated and/or analyzed during the current study are available in the (figshare) repository (https://figshare.com/articles/MP_readmission/6838043). The data showed 424 cases with readmission and index hospitalization after MPP.

## Abbreviations

CAP: Community acquired pneumonia
GeXP assay: GenomeLab Genetic Analysis System
*MP or M. pneumoniae*: *Mycoplasma pneumoniae*
RT-PCR: Reverse transcription polymerase chain reaction

## References

[1] Tang VL, Halm EA, Fine MJ, Johnson CS, Anzueto A, Mortensen EM (2014). Predictors of rehospitalization after admission for pneumonia in the veterans affairs healthcare system. J Hosp Med.

[2] Toledo D, Soldevila N, Torner N, Perez-Lozano MJ, Espejo E, Navarro G, Egurrola M, Dominguez A (2018). On-behalf of the project FISPIWG: factors associated with 30-day readmission after hospitalisation for community-acquired pneumonia in older patients: a cross-sectional study in seven Spanish regions. BMJ Open.

[3] Torres A, Peetermans WE, Viegi G, Blasi F (2013). Risk factors for community-acquired pneumonia in adults in Europe: a literature review. Thorax.

[4] Capelastegui A, Espana Yandiola PP, Quintana JM, Bilbao A, Diez R, Pascual S, Pulido E, Egurrola M (2009). Predictors of short-term rehospitalization following discharge of patients hospitalized with community-acquired pneumonia. Chest.

[5] Dong T, Cursio JF, Qadir S, Lindenauer PK, Ruhnke GW (2017). Discharge disposition as an independent predictor of readmission among patients hospitalised for community-acquired pneumonia. Int J Clin Pract.

[6] Jasti H, Mortensen EM, Obrosky DS, Kapoor WN, Fine MJ (2008). Causes and risk factors for rehospitalization of patients hospitalized with community-acquired pneumonia. Clin Infect Dis.

[7] Adamuz J, Viasus D, Camprecios-Rodriguez P, Canavate-Jurado O, Jimenez-Martinez E, Isla P, Garcia-Vidal C, Carratala J (2011). A prospective cohort study of healthcare visits and rehospitalizations after discharge of patients with community-acquired pneumonia. Respirology.

[8] Brogan TV, Hall M, Williams DJ, Neuman MI, Grijalva CG, Farris RW, Shah SS (2012). Variability in processes of care and outcomes among children hospitalized with community-acquired pneumonia. Pediatr Infect Dis J.

[9] Neuman MI, Hall M, Gay JC, Blaschke AJ, Williams DJ, Parikh K, Hersh AL, Brogan TV, Gerber JS, Grijalva CG (2014). Readmissions among children previously hospitalized with pneumonia. Pediatrics.

[10] Nakamura MM, Zaslavsky AM, Toomey SL, Petty CR, Bryant MC, Geanacopoulos AT, Jha AK, Schuster MA (2017). Pediatric Readmissions After Hospitalizations for Lower Respiratory Infections. Pediatrics.

[11] Berry JG, Toomey SL, Zaslavsky AM, Jha AK, Nakamura MM, Klein DJ, Feng JY, Shulman S, Chiang VW, Kaplan W (2013). Pediatric readmission prevalence and variability across hospitals. Jama.

[12] Lee KY (2008). Pediatric respiratory infections by mycoplasma pneumoniae. Expert Rev Anti-Infect Ther.

[13] Nelson S, Belknap SM, Carlson RW, Dale D, DeBoisblanc B, Farkas S, Fotheringham N, Ho H, Marrie T, Movahhed H (1998). A randomized controlled trial of filgrastim as an adjunct to antibiotics for treatment of hospitalized patients with community-acquired pneumonia. CAP study group. J Infect Dis.

[14] Barker CE, Sillis M, Wreghitt TG (1990). Evaluation of Serodia Myco II particle agglutination test for detecting mycoplasma pneumoniae antibody: comparison with mu-capture ELISA and indirect immunofluorescence. J Clin Pathol.

[15] Templeton KE, Scheltinga SA, Graffelman AW, Van Schie JM, Crielaard JW, Sillekens P, Van Den Broek PJ, Goossens H, Beersma MF, Claas EC (2003). Comparison and evaluation of real-time PCR, real-time nucleic acid sequence-based amplification, conventional PCR, and serology for diagnosis of mycoplasma pneumoniae. J Clin Microbiol.

[16] Suzuki S, Yamazaki T, Narita M, Okazaki N, Suzuki I, Andoh T, Matsuoka M, Kenri T, Arakawa Y, Sasaki T (2006). Clinical evaluation of macrolide-resistant mycoplasma pneumoniae. Antimicrob Agents Chemother.

[17] Wang L, Feng Z, Zhao M, Yang S, Yan X, Guo W, Shi Z, Li G (2017). A comparison study between GeXP-based multiplex-PCR and serology assay for mycoplasma pneumoniae detection in children with community acquired pneumonia. BMC Infect Dis.

[18] Wang L, Zhao M, Shi Z, Feng Z, Guo W, Yang S, Liu L, Li G (2016). A GeXP-based assay for simultaneous detection of multiple viruses in hospitalized children with community acquired pneumonia. PLoS One.

[19] Driscoll AJ, Karron RA, Morpeth SC, Bhat N, Levine OS, Baggett HC, Brooks WA, Feikin DR, Hammitt LL, Howie SRC (2017). Standardization of Laboratory Methods for the PERCH Study. Clin Infect Dis.

[20] Marks C, Loehrer S, McCarthy D (2013). Hospital readmissions: measuring for improvement, accountability, and patients. Issue brief.

[21] Andruska A, Micek ST, Shindo Y, Hampton N, Colona B, McCormick S, Kollef MH (2015). Pneumonia pathogen characterization is an independent determinant of hospital readmission. Chest.

[22] Toomey SL, Peltz A, Loren S, Tracy M, Williams K, Pengeroth L, Ste Marie A, Onorato S, Schuster MA (2016). Potentially Preventable 30-Day Hospital Readmissions at a Children's Hospital. Pediatrics.

[23] Waites KB, Talkington DF (2004). Mycoplasma pneumoniae and its role as a human pathogen. Clin Microbiol Rev.

[24] Williams DJ, Hall M, Brogan TV, Farris RW, Myers AL, Newland JG, Shah SS (2011). Influenza coinfection and outcomes in children with complicated pneumonia. Arch Pediatr Adolesc Med.

[25] Brogan TV, Hall M, Sills MR, Fieldston ES, Simon HK, Mundorff MB, Fagbuyi DB, Shah SS (2014). Hospital readmissions among children with H1N1 influenza infection. Hosp Pediatr.

[26] Halm EA, Fine MJ, Kapoor WN, Singer DE, Marrie TJ, Siu AL (2002). Instability on hospital discharge and the risk of adverse outcomes in patients with pneumonia. Arch Intern Med.

[27] Auerbach AD, Kripalani S, Vasilevskis EE, Sehgal N, Lindenauer PK, Metlay JP, Fletcher G, Ruhnke GW, Flanders SA, Kim C (2016). Preventability and causes of readmissions in a National Cohort of general medicine patients. JAMA Intern Med.

[28] Gay JC, Hain PD, Grantham JA, Saville BR (2011). Epidemiology of 15-day readmissions to a Children's hospital. Pediatrics.

